# Estimating the percentage of patients who might benefit from proton beam therapy instead of X-ray radiotherapy

**DOI:** 10.1259/bjr.20211175

**Published:** 2022-03-17

**Authors:** Neil G Burnet, Thomas Mee, Simona Gaito, Norman F Kirkby, Adam H Aitkenhead, Carmel N Anandadas, Marianne C Aznar, Lisa H Barraclough, Gerben Borst, Frances C Charlwood, Matthew Clarke, Rovel J Colaco, Adrian M Crellin, Noemie N Defourney, Christina J Hague, Margaret Harris, Nicholas T Henthorn, Kirsten I Hopkins, E Hwang, Sam P Ingram, Karen J Kirkby, Lip W Lee, David Lines, Zoe Lingard, Matthew Lowe, Ranald I Mackay, Catherine A McBain, Michael J Merchant, David J Noble, Shermaine Pan, James M Price, Ganesh Radhakrishna, David Reboredo-Gil, Ahmed Salem, Srijith Sashidharan, Peter Sitch, Ed Smith, Edward AK Smith, Michael J Taylor, David J Thomson, Nicola J Thorp, Tracy SA Underwood, John W Warmenhoven, James P Wylie, Gillian Whitfield

**Affiliations:** The Christie NHS Foundation Trust, Wilmslow Rd, Manchester, United Kingdom; Division of Cancer Sciences, University of Manchester, Manchester Cancer Research Centre, Manchester Academic Health Science Centre, Manchester, United Kingdom; The Christie NHS Foundation Trust, Wilmslow Rd, Manchester, United Kingdom; Division of Cancer Sciences, University of Manchester, Manchester Cancer Research Centre, Manchester Academic Health Science Centre, Manchester, United Kingdom; Division of Cancer Sciences, University of Manchester, Manchester Cancer Research Centre, Manchester Academic Health Science Centre, Manchester, United Kingdom; Division of Cancer Sciences, University of Manchester, Manchester Cancer Research Centre, Manchester Academic Health Science Centre, Manchester, United Kingdom; Christie Medical Physics and Engineering, The Christie NHS Foundation Trust, Wilmslow Road, Manchester, United Kingdom; The Christie NHS Foundation Trust, Wilmslow Rd, Manchester, United Kingdom; The Christie NHS Foundation Trust, Wilmslow Rd, Manchester, United Kingdom; Division of Cancer Sciences, University of Manchester, Manchester Cancer Research Centre, Manchester Academic Health Science Centre, Manchester, United Kingdom; The Christie NHS Foundation Trust, Wilmslow Rd, Manchester, United Kingdom; The Christie NHS Foundation Trust, Wilmslow Rd, Manchester, United Kingdom; Division of Cancer Sciences, University of Manchester, Manchester Cancer Research Centre, Manchester Academic Health Science Centre, Manchester, United Kingdom; Christie Medical Physics and Engineering, The Christie NHS Foundation Trust, Wilmslow Road, Manchester, United Kingdom; Christie Medical Physics and Engineering, The Christie NHS Foundation Trust, Wilmslow Road, Manchester, United Kingdom; The Christie NHS Foundation Trust, Wilmslow Rd, Manchester, United Kingdom; Division of Cancer Sciences, University of Manchester, Manchester Cancer Research Centre, Manchester Academic Health Science Centre, Manchester, United Kingdom; NHS England National Clinical Lead Proton Beam Therapy, Leeds Cancer Centre, Leeds Teaching Hospitals NHS Trust, Leeds and St James's Institute of Oncology, Leeds Teaching Hospitals NHS Trust, Beckett Street, Leeds, LS9 7TF, UK, Leeds, United Kingdom; Division of Cancer Sciences, University of Manchester, Manchester Cancer Research Centre, Manchester Academic Health Science Centre, Manchester, United Kingdom; The Christie NHS Foundation Trust, Wilmslow Rd, Manchester, United Kingdom; The Christie NHS Foundation Trust, Wilmslow Rd, Manchester, United Kingdom; Division of Cancer Sciences, University of Manchester, Manchester Cancer Research Centre, Manchester Academic Health Science Centre, Manchester, United Kingdom; International Atomic Energy Agency, Vienna International Centre, Vienna, Austria; The Christie NHS Foundation Trust, Wilmslow Rd, Manchester, United Kingdom; Department of Radiation Oncology, Sydney West Radiation Oncology Network, Crown Princess Mary Cancer Centre, Sydney, New South Wales, Australia and Institute of Medical Physics, School of Physics, University of Sydney, Sydney, New South Wales, Australia; Division of Cancer Sciences, University of Manchester, Manchester Cancer Research Centre, Manchester Academic Health Science Centre, Manchester, United Kingdom; Christie Medical Physics and Engineering, The Christie NHS Foundation Trust, Wilmslow Road, Manchester, United Kingdom; Division of Cancer Sciences, University of Manchester, Manchester Cancer Research Centre, Manchester Academic Health Science Centre, Manchester, United Kingdom; The Christie NHS Foundation Trust, Wilmslow Rd, Manchester, United Kingdom; Christie Medical Physics and Engineering, The Christie NHS Foundation Trust, Wilmslow Road, Manchester, United Kingdom; Division of Cancer Sciences, University of Manchester, Manchester Cancer Research Centre, Manchester Academic Health Science Centre, Manchester, United Kingdom; Division of Cancer Sciences, University of Manchester, Manchester Cancer Research Centre, Manchester Academic Health Science Centre, Manchester, United Kingdom; Christie Medical Physics and Engineering, The Christie NHS Foundation Trust, Wilmslow Road, Manchester, United Kingdom; Christie Medical Physics and Engineering, The Christie NHS Foundation Trust, Wilmslow Road, Manchester, United Kingdom; The Christie NHS Foundation Trust, Wilmslow Rd, Manchester, United Kingdom; Division of Cancer Sciences, University of Manchester, Manchester Cancer Research Centre, Manchester Academic Health Science Centre, Manchester, United Kingdom; Department of Clinical Oncology, Edinburgh Cancer Centre, Western General Hospital, Edinburgh, United Kingdom; Division of Cancer Sciences, University of Manchester, Manchester Cancer Research Centre, Manchester Academic Health Science Centre, Manchester, United Kingdom; The Christie NHS Foundation Trust, Wilmslow Rd, Manchester, United Kingdom; Division of Cancer Sciences, University of Manchester, Manchester Cancer Research Centre, Manchester Academic Health Science Centre, Manchester, United Kingdom; The Christie NHS Foundation Trust, Wilmslow Rd, Manchester, United Kingdom; Christie Medical Physics and Engineering, The Christie NHS Foundation Trust, Wilmslow Road, Manchester, United Kingdom; The Christie NHS Foundation Trust, Wilmslow Rd, Manchester, United Kingdom; Division of Cancer Sciences, University of Manchester, Manchester Cancer Research Centre, Manchester Academic Health Science Centre, Manchester, United Kingdom; The Christie NHS Foundation Trust, Wilmslow Rd, Manchester, United Kingdom; Christie Medical Physics and Engineering, The Christie NHS Foundation Trust, Wilmslow Road, Manchester, United Kingdom; The Christie NHS Foundation Trust, Wilmslow Rd, Manchester, United Kingdom; Proton Clinical Outcomes Unit, The Christie NHS Foundation Trust, Manchester, United Kingdom; Division of Cancer Sciences, University of Manchester, Manchester Cancer Research Centre, Manchester Academic Health Science Centre, Manchester, United Kingdom; Christie Medical Physics and Engineering, The Christie NHS Foundation Trust, Wilmslow Road, Manchester, United Kingdom; Division of Cancer Sciences, University of Manchester, Manchester Cancer Research Centre, Manchester Academic Health Science Centre, Manchester, United Kingdom; The Christie NHS Foundation Trust, Wilmslow Rd, Manchester, United Kingdom; Division of Cancer Sciences, University of Manchester, Manchester Cancer Research Centre, Manchester Academic Health Science Centre, Manchester, United Kingdom; The Christie NHS Foundation Trust, Wilmslow Rd, Manchester, United Kingdom; Division of Cancer Sciences, University of Manchester, Manchester Cancer Research Centre, Manchester Academic Health Science Centre, Manchester, United Kingdom; Division of Cancer Sciences, University of Manchester, Manchester Cancer Research Centre, Manchester Academic Health Science Centre, Manchester, United Kingdom; The Christie NHS Foundation Trust, Wilmslow Rd, Manchester, United Kingdom; The Christie NHS Foundation Trust, Wilmslow Rd, Manchester, United Kingdom; Division of Cancer Sciences, University of Manchester, Manchester Cancer Research Centre, Manchester Academic Health Science Centre, Manchester, United Kingdom

## Abstract

**Objectives:**

High-energy Proton Beam Therapy (PBT) commenced in England in 2018 and NHS England commissions PBT for 1.5% of patients receiving radical radiotherapy. We sought expert opinion on the level of provision.

**Methods:**

Invitations were sent to 41 colleagues working in PBT, most at one UK centre, to contribute by completing a spreadsheet. 39 responded: 23 (59%) completed the spreadsheet; 16 (41%) declined, arguing that clinical outcome data are lacking, but joined six additional site-specialist oncologists for two consensus meetings. The spreadsheet was pre-populated with incidence data from Cancer Research UK and radiotherapy use data from the National Cancer Registration and Analysis Service. ‘Mechanisms of Benefit’ of reduced growth impairment, reduced toxicity, dose escalation and reduced second cancer risk were examined.

**Results:**

The most reliable figure for percentage of radical radiotherapy patients likely to benefit from PBT was that agreed by 95% of the 23 respondents at 4.3%, slightly larger than current provision. The median was 15% (range 4–92%) and consensus median 13%. The biggest estimated potential benefit was from reducing toxicity, median benefit to 15% (range 4–92%), followed by dose escalation median 3% (range 0 to 47%); consensus values were 12 and 3%. Reduced growth impairment and reduced second cancer risk were calculated to benefit 0.5% and 0.1%.

**Conclusions:**

The most secure estimate of percentage benefit was 4.3% but insufficient clinical outcome data exist for confident estimates. The study supports the NHS approach of using the evidence base and developing it through randomised trials, non-randomised studies and outcomes tracking.

**Advances in knowledge:**

Less is known about the percentage of patients who may benefit from PBT than is generally acknowledged. Expert opinion varies widely. Insufficient clinical outcome data exist to provide robust estimates. Considerable further work is needed to address this, including international collaboration; much is already underway but will take time to provide mature data.

## Introduction

High-energy Proton Beam Therapy (PBT) commenced in England in 2018, with the opening of the Proton Beam Therapy Centre at The Christie NHS Foundation Trust (The Christie) in Manchester; the second NHS centre, at University College London Hospitals NHS Foundation Trust (UCLH), opened in late 2021. This followed 10 years of provision under the Proton Overseas Programme (POP), which by then had provided treatment abroad to over 1100 patients, with costs met by the NHS.^1–3^

Currently, NHS England commissions PBT for curative intent, using an evidence-based approach, with emphasis on children, young adults under 25 years old, and adults with rare tumours that benefit from dose escalation^2,4^ (see Appendix 1 in^4^). Although there is clear consensus that PBT has a role in radiotherapy (RT), there is no agreement on what percentage of patients might benefit from it. Overall, the 2 NHS PBT centres have capacity to treat about 1.5% of patients receiving radical RT, including capacity for patients in the clinical evaluation programmes.^2^ Other countries are suggesting that a higher rate of PBT provision might be beneficial, most in the range 3.5–15%,^1,2,5,6^ and there are even higher, older estimates (~30%).^7^

The major perceived value of PBT is to reduce toxicity, especially valuable for children, with reduced effects on growth, function and risk of second malignant neoplasm (SMN). In adults, PBT appears to offer an advantage for tumours such as chordoma of the skull base and spine by facilitating modest dose escalation; these are challenging to manage but few in number.^8^ For most other adults, evidence of clinically meaningful benefit from PBT rather than intensity-modulated (X-ray) radiotherapy (IMRT), and the magnitude of any such benefit, has not yet been demonstrated,^9–13^ although trials are ongoing.

The major challenge with estimating the percentage of radical RT patients who might benefit from PBT is the relative lack of clinical data, leading to reliance on dosimetry differences between PBT and X-ray radiotherapy (XRT). This may, but does not necessarily, translate into differences in clinical outcome. To address this, randomised-controlled trials (RCTs) have a crucial place but cannot address all the relevant questions.^14–16^ Additional methodological approaches are needed, including Evaluative Commissioning and Outcomes Tracking.^4,14,17–19^ The latter approach is more powerful than often appreciated and provides for long-term follow-up, which is relevant for a number of patient-centred endpoints.

Conscious of the relative lack of evidence, we sought expert opinion to address the question of what percentage of patients might be considered likely to benefit from PBT.

## Methods

**Figure 1.**
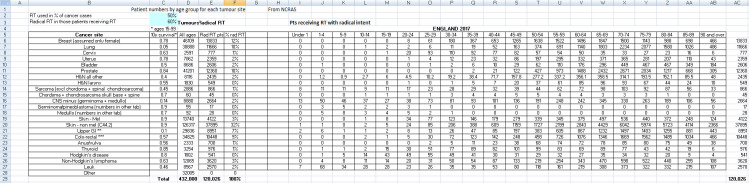
The core data ‘tab’ showing main tumour sites, estimated 10-year survival, total patient numbers, numbers estimated to receive curative RT, the percentage of all curative RT for each site and how these patients are distributed across the age group categories of 5 years. The oldest category is ‘Age 90 and over’. Children were defined as 16 and under, with numbers interpolated within the 15–19 years age category. Note that the generic assumptions of percentages of (1) cancer patients receiving any RT (50%) and (2) the percentage treated with curative intent (60%) are shown at the top left (Cells C2 & C3, blue). In this scenario, the radical RT numbers are dominated by non-melanoma skin cancer (32%, 37895 patients), breast (12%), lung (10%) and prostate (10%). To use the NCRAS site-by-site data, the specific tumour RT data were entered into Columns E & F site-by-site, the dependency to Cells C2 and C3 was removed, and the numbers in each age category were scaled. Note that the total cases per annum receiving radical RT shown here with the generic assumptions (50%, 60% in cells C2 & C3) is 120,026 while the actual NCRAS data show that only 86064 patients received radical RT (see Table 2). H&N = Head and Neck, medullo = medulloblastoma, Mel = melanoma, GI = gastro-intestinal, Leuk = leukaemia.

A spreadsheet was developed to address each of four established ‘Mechanisms of Benefit’ (MoBs) in which PBT may provide clinical benefit (Table 1), pre-populated with incidence data from the Office of National Statistics (ONS) for England (Figure 1),^20^ data on use of curative RT from the National Cancer Registration and Analysis Service (NCRAS) taken from the Radiotherapy Dataset (RTDS) on radiotherapy activity in England^21^ and survival data from Cancer Research UK.^22^ See Supplementary Material for full details.

**Table 1.** The four main established ‘Mechanisms of Benefit’ (MoBs) through which PBT, compared to X-ray therapy, may deliver a clinical advantage to patients. Growth impairment is clearly a form of normal tissue toxicity, which is specifically applicable to growing children.

1	Growth impairment
2	Dysfunction of and toxicity in normal tissues
3	Dose escalation
4	Reduction in second cancer risk.

**Table 2.** Estimates of the percentage (and number) of patients who might benefit from PBT, assuming patients counted only once, with estimates of the absolute numbers, for different values of the RT utilisation parameters (percentage of cancer cases receiving RT and the percentage of RT cases treated with curative (radical) intent). See Supplementary Material for more details. The most accurate estimates should be from the site-by-site NCRAS data. Note that the number of radical RT patients calculated using the ‘Traditional’ generic data is substantially different from the number derived from the more accurate NCRAS data. However, estimates of the number of patients estimated to benefit from the consensus are almost the same using ‘Traditional’ generic and NCRAS data, although the percentages are different, 9% versus 13%.

Assumptions	Individual participants^*a*^			Consensus	
	‘Traditional’^*b*^	Generic NCRAS data	Site-by-site NCRAS data	‘Traditional’	Site-by-site NCRAS data
% receiving any RT	50%	31%^*c*^	-	50%	-
% radical RT	60%	70%^*c*^	-	60%	-
Total expected					
Radical RT patients (% change compared to ‘Traditional’)	120026	86831 (−28%)	86064^*d*^ (−28%)	120026	86064^*d*^ (−28%)
% (number) of patients estimated as likely to benefit from PBT					
Consensus value				9% (10447)	13% (10905)
Median	12% (14945)	12% (10823)	15%(12941)		
Mean	19% (23391)	19% (16930)	24%(20903)		
Max	92% (110545)	92% (79973)	92% (79415)		
Min	3% (3194)	3% (2320)	4% (3445)		

a n=23 participants.

b Burnet et al. BMJ 2000, IARC 2011.

c Calculated from the site-by-site data, excluding ‘Other’ site patients.

d Calculated from NCRAS site-by-site data of total percentages of patients having RT x those receiving radical RT.

### Participants

#### Individual participants

During the second half of 2020, invitations to complete the spreadsheet were sent to 41 colleagues (clinical oncologists, medical physicists and proton research scientists), 39 working in PBT at The Christie PBT Centre or University of Manchester, and two from elsewhere with a long-standing interest and a UK focus (see Supplementary Material).

**Figure 2.**
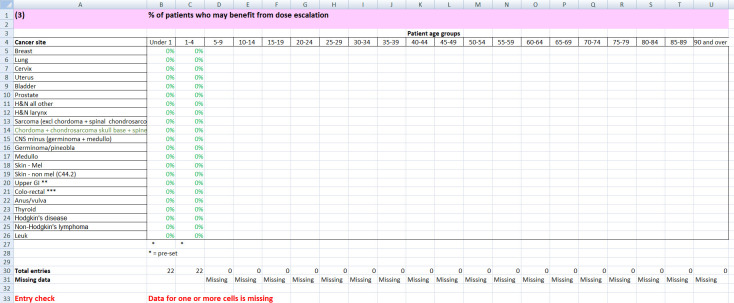
Dose escalation spreadsheet ‘tab’ that participants were asked to complete. Note that the columns for ages ‘Under 1’ and ‘1-4’ were pre-populated with 0%. Participants did not need to know patient numbers in each cell since this was pre-set in the spreadsheet, from the Core Data tab, shown in Figure 1.

Participants were invited to estimate the percentage of patients within each tumour type and age category who might benefit from MoBs 2 and 3 by completing two worksheet ‘tabs’ (Figure 2). ‘Benefit’ was explicitly defined qualitatively, as ‘considered likely to confer clinical advantage to the patient’. This qualitative definition was used in order to avoid the need to specify scoring tools, each with a semi-arbitrary defined level of toxicity, for each specific endpoint, in each normal tissue.

The magnitude of possible benefit was not addressed.

Two consensus meetings were held to provide a group perspective. Six additional oncologists were recruited to complete specific tumour-site rows of the spreadsheet for the consensus.

### Assumptions on the use of RT

Generic assumptions on (1) percentage of cancer cases receiving RT and (2) the percentage of RT cases treated with curative intent are often made in order to calculate numbers requiring treatment. Therefore, analysis was performed with such assumptions applied uniformly to each tumour, using ‘Traditional’ values^23,24^ and values derived from the NCRAS data set. The site-specific NCRAS data were then used (Table 2).

### Mechanisms of benefit (MoBs)

Participants entered data for MoBs 2 and 3; the spreadsheet automatically calculated the patients potentially benefitting from MoBs 1 and 4 (see Supplementary Material). The overall percentage of patients who might benefit was estimated by counting each patient only once, even if potentially benefitting from more than one MoB.

## Results

### Individual participants

**Figure 3.**
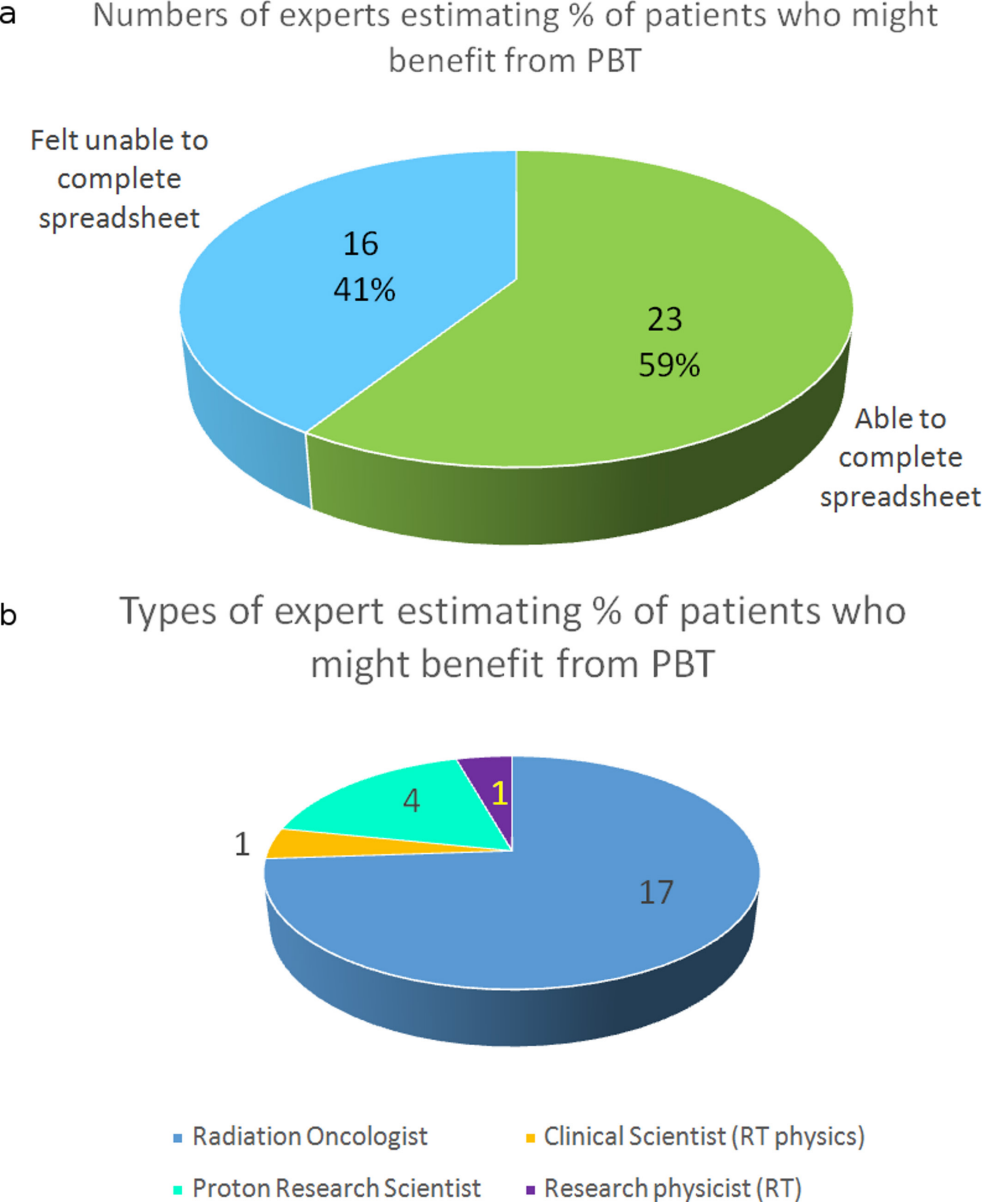
a. Numbers of participants who felt able (59%) to complete the spreadsheet or unable (41%) because they felt there was insufficient clinical information to make reasoned recommendations. b. Distribution of professional roles in the 23 participants who completed the full spreadsheet. The professional roles of the remaining 16 were: radiation (clinical) oncologists 2, clinical scientists in radiotherapy physics 9 and proton research scientists 5.

Of the 41 invitees, 39 (95%) responded. Of these, 23 (59%) completed the spreadsheet (Figure 3). A further 16 (41%) declined (Figure 3), arguing that there is too little clinical outcome data available to provide for robust estimates, that what there is can be inconsistent, and several were unwilling to contribute wild guesses. All expressed uncertainty about their responses and the clinicians identified site specialisation as reducing their confidence about other sites.

All participants made assumptions about change of benefit with age: many assumed reducing benefit with increasing age, some assumed the opposite, and some assumed first reducing benefit then increasing advantage with age. This underlines the relative lack of hard data to populate predictions of benefit.

Some respondents noted that there is a paucity of information on dose response for many tumours, which makes estimating dose escalation difficult. No instructions were given in the initial invitations but at the consensus meetings it was explicitly stated that motion management should be assumed to be available for lung, chest and abdominal tumours, since work on solutions to this difficult problem had started. Intrafraction motion especially from breathing can result in dose uncertainties particularly in regions with large density heterogeneities, such as the lung. Motion management techniques, such as gating, breath-hold delivery and abdominal compression, can help to minimise motion during treatment delivery, and reducing in-field dose gradients can help to reduce the effect of residual motion on delivered dose.

It was agreed that knowledge of the percentages of patients who suffer from side-effects from XRT would be useful. Although excellent reviews are available (*e.g.,* ^9^), some data are not available and much relates to the pre-image-guided IMRT era. Therefore the qualitative approach was used to estimate ‘clinical advantage to the patient’.

### Estimates of overall benefit

**Figure 4.**
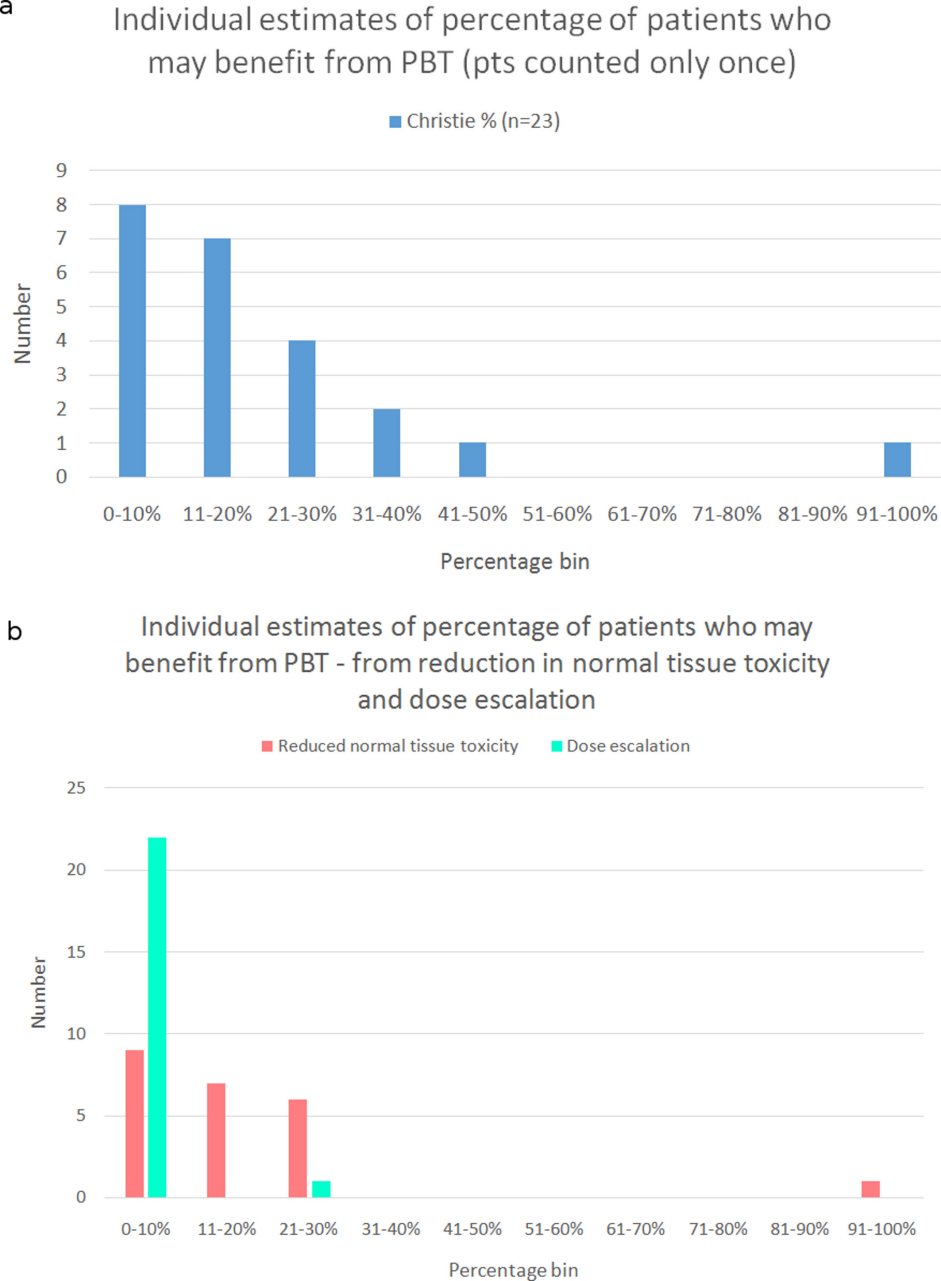
a. Distribution of individual responses for overall percentage of patients who might benefit from PBT, counting patients only once and calculated from NCRAS site-by-site data (the graph for generic RT usage (50% receive RT, 60% radical) is identical). One outlier estimated that 92% might benefit. Although almost two-thirds (65%) of participants estimated the overall percentage of patients who might benefit to be between 0 and 20%, the minimum figure estimated by 95% of participants was 4.3%. b. Distribution of individual responses for potential benefit from MoB 2, ‘Reduction in dysfunction and toxicity in normal tissues’, and from MoB 3, ‘Dose escalation’. One participant estimated that a very high percentage (92%) of patients might benefit as a result of a very high estimate of gain from reduction in normal tissue toxicity (MoB 2). A different participant estimated a high benefit (35%) from dose escalation (MoB 3). This was the only participant who prioritised dose escalation ahead of reduction in normal tissue toxicity.

Counting patients only once, with generic global values of RT use, the 23 completed spreadsheets estimated a median 12% of patients considered likely to benefit, range 3–92%, (Table 2). Using the NCRAS site-by-site RT data, the median value was slightly higher at 15%, range 4–92%. The overall minimum percentage estimate from 95% of the participants was 4.3%, which may be a figure with lower uncertainty. The distribution of individual responses (Figure 4a) is highly skewed.

The two consensus meetings estimated 9% of patients likely to benefit, using generic values for RT use, and 13% using the more accurate NCRAS site-specific RT use data (Table 2). However, the absolute number of cases was very similar.

There was no statistically significant difference between oncologists (*n* = 17) and non-oncologists (*n* = 6) (t test, 2-tailed, *p* = 0.87).

### Estimates of benefit from each MoB

Results for each MoB individually are shown in Table 3. The biggest potential benefit, and the biggest range in individual estimates (*n* = 23), was for MoB 2. Using the NCRAS site-by-site RT data, these values are median 15%, range 4–92%. For MoB 3, the median percentage estimated to benefit was 3%, range 0–47%. These distributions are also skewed (Figure 4).

**Table 3.** Estimates of the percentage of patients who might benefit from each MoB individually, based on both individual participants and the Consensus. MoBs 1 and 4 are calculated automatically; they have no participant input and so have no range.

Individual participants^*a*^ ^*b*^										
MoB	RT use - ‘Traditional’					RT use – NCRAS site-by-site				
	Median	Mean	Max	Min	Calculated Value	Median	Mean	Max	Min	Calculated Value
Growth impairment					0.4%					0.5%
Dysfunction of and toxicity in normal tissues	12%	18%	92%	2%		15%	23%	92%	4%	
Dose escalation	3%	5%	35%	0		3%	6%	47%	0	
Reduction in second cancer risk					0.1%					0.1%

Consensus^*b*^				
MoB	RT use - ‘Traditional’		RT use – NCRAS site-by-site	
	Consensus values		Consensus values	
	Estimate	Calculated value	Estimate	Calculated
Growth impairment		0.4%		0.5%
Dysfunction of and toxicity in normal tissues	7%		12%	
Dose escalation	3%		3%	
Reduction in second cancer risk		0.1%		0.1%

a n=23 participants.

b Note that since some patients could benefit from more than one MoB, percentages may total more than 100%.

From the consensus, the biggest predicted benefit was also from MoB 2, 12% with NCRAS site-specific data (Table 3). This is smaller than the median value for the individual contributors, although 3% for MoB 3 is the same. The tumour sites judged most likely to express benefit from MoBs 2 and 3 are shown in Table 4.

**Table 4.** Estimates of the number of patients who might benefit from MoBs 2 and 3, showing the distribution by tumour site (Consensus estimates, NCRAS site-by-site RT data). Column 2 in each block shows the percentage distribution of benefit for that specific MoB; Column 3 in each block shows the numbers as a percentage of all radical RT cases. Cancer sites that make up 5% or more of those who may benefit are shown in green. In MoB 2 (reducing normal tissue toxicity), the biggest estimated advantage is for HNC, followed by CNS. In MoB 3 (dose escalation), the biggest estimated advantage is for lung cancer, followed by HNC. It is important to note that the total numbers of cases play an important part in this ranking. For example, chordoma and chondrosarcoma of the skull base and spine are rare but robustly evidence-based and considered strong indications for PBT but being rare are not highlighted here.

Number of radical RT cases	Tumour site	(2) Normal tissue function preservation			(3) Advantage from dose escalation		
		Number benefitting	% of MOB2	% of total radical RT	Number benefitting	% of MOB3	% of total radical RT
							
30466	Breast	464	4.7%	1%	232	9%	0%
6266	Lung	505	5.1%	1%	1092	44%	1%
1409	Cervix	412	4%	0%	0	0%	0%
3269	Uterus	306	3%	0%	0	0%	0%
1249	Bladder	6	0%	0%	38	2%	0%
17098	Prostate	28	0%	0%	281	11%	0%
4971	H&N all other	4531	46%	5%	495	20%	1%
1121	H&N larynx	4	0%	0%	0	0%	0%
971	Sarcoma (excl chordoma + spinal chondrosa)	605	6%	1%	86	3%	0%
57	Chordoma + chondrosa skull base + spine	57	1%	0%	57	2%	0%
3376	CNS minus (germinoma + medullo)	1371	14%	2%	143	6%	0%
51	Germinoma/pineobla	50	1%	0%	0	0%	0%
86	Medullo	86	1%	0%	0	0%	0%
434	Skin - Mel	1	0%	0%	0	0%	0%
4820	Skin - non mel (C44.2)	1	0%	0%	0	0%	0%
1615	Upper GI **	441	4%	1%	45	2%	0%
4026	Colo-rectal ***	1	0%	0%	0	0%	0%
990	Anus/vulva	655	7%	1%	0	0%	0%
281	Thyroid	35	0%	0%	9	0%	0%
951	Hodgkin's disease	219	2%	0%	0	0%	0%
2311	Non-Hodgkin's lymphoma	108	1%	0%	0	0%	0%
247	Leuk	0	0%	0%	0	0%	0%
	Other						
86064		9885	100%	12%	2479	100%	3%

MoB 1 is estimated to provide benefit to 0.5% of patients, MoB 4 just 0.1%. These are calculated automatically and therefore have no range.

### Differences resulting from assumed levels of RT use

The percentages of patients estimated as likely to benefit are the same for any generic assumptions of RT use (Table 2), although the estimated absolute numbers of patients are substantially different (Table 2). However, the overall use of RT from the NCRAS data is quite low at 31%, and may not be ideal. Clearly provision of a national service is dependent on the actual number of patients needing treatment, rather than a percentage.

### Differences resulting from changes in assumptions for SMN risk

The assumption for latency for SMN risk after RT (15 years) and the scaling of the estimates of risk from radiation exposure (1.9) were varied to assess their impact. For both, variation allowing for greater numbers of SMN altered the predicted percentages from 0.1 to 0.2%. Thus, within the limits of this methodology, altering the exact values of these variables produces little difference.

## Discussion

Although PBT has a definite place in RT, there is no consensus on the extent of its role.^8^ This has important implications for the provision of facilities and manpower, and for forward planning; it also has a politico-economic perspective. There are additional challenges in quantifying the magnitude of benefit and agreeing what magnitude of benefit should be considered cost-effective.

This has been a topic of interest for many years^7,25,26^ and the number of patients considered likely to benefit has mostly reduced over time.^16^ This is especially the result of huge developments in the quality of conventional XRT, particularly image-guided IMRT, stereotactic ablative radiotherapy (SABR) and radiosurgery. Additionally, in areas particularly relevant to potential PBT indications, such as prostate radiotherapy, the use of pre-rectal spacers and watch-and-wait strategies have changed the climate dramatically. Nevertheless, PBT indications are now being expanded in some European centres,^8^ due partly to comparative planning with normal tissue complication probability (NTCP) calculations to select patients on an individual basis^27^ and partly from other factors less easy to define. Similar discussions are now occurring about carbon ion therapy.^28^

### Estimates of overall benefit

A striking finding of our study was that 41% of participants actively declined to complete the spreadsheet because they felt there are too few clinical outcome data for many tumour types and age groups.

From the individual participants, the overall estimate of percentage of patients likely to benefit was a median of 15%. However, there was a huge individual range (Tables 2 and 3, Figure 4), which emphasises the uncertainty in estimates of potential PBT benefit. 65% of participants estimated the percentage as between 0 and 20%. However, the estimate including 95% of participants was 4.3%. This figure, derived from the distribution of responses, is likely to be a figure with lower uncertainty than simple median values.

The consensus estimate of likely benefit from PBT was 13%. However, it is likely to be an overestimate, perhaps a substantial one. Many of the interactions at the two meetings involved enthusiasts being persuaded by other less optimistic enthusiasts that the real potential was likely to be less. The fact that participants were largely from one UK centre might introduce some bias, particularly since the length of clinical experience is comparatively short. The discrepancies between the modest existing clinical data and the tumour site predictions (Table 4) also imply an overoptimistic perception. This 13% figure is substantially higher than the existing NHS England provision, and it remains unproven whether this is a clinically realistic figure.

**Figure 5.**
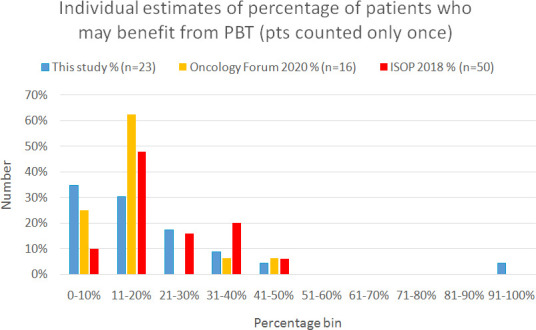
Distribution of individual responses for the study presented here (n=23) compared to opinion results from the International Symposium on Proton Therapy (ISOP) held in Heidelberg in 2018 (results for adults only; expert international audience) (n=50) and for the Oncology Forum conference, held on-line in 2020 (non-expert oncology audience) (n=16). Note that children (in the NCRAS site-by-site dataset) account for only 0.5% of cases. Median for the individual participants in our study was 15%, while the consensus figure was 13%. Approximate medians for ISOP and the Oncology Forum were both 15%, with the majority estimating likely benefit from PBT ≤20%. The three data sets are quite similar although there may be differences between countries in the perceived potential benefit, manifest in the generally higher estimates shown in the ISOP results.

These results can be informally compared with (unpublished) results of polls of participants at the 2018 International Symposium on Proton Therapy (ISOP) and the 2020 Oncology Forum (Figure 5). This illustrates that uncertainty is typical.

### Estimates of benefit from each mechanism of benefit (MoB)

This can indicate where clinical studies could be focussed. Estimates suggest that most benefit will occur from MoB 2, reduction in toxicity (12%), followed by MoB 3, dose escalation (3%) (Table 3). The range of estimates is large, especially for MoB 2 (Table 3, Figure 4).

The sites estimated most likely to benefit are shown in Table 4. Head and neck cancer (HNC) is considered to benefit most from both MoB 2 and 3. It is no coincidence that the TORPEdO trial^29^ is evaluating PBT in oropharynx cancer nor that HNC has been a major focus of the Dutch NTCP modelling approach.^27,30,31^ Both CNS and lung cancer are also identified as potentially benefitting large numbers of patients, with more than 1000 patients estimated to benefit, from MoB 2 and MoB 3, respectively. However, clinical evidence is lacking for CNS^9,11^ and current literature suggests no advantage from conventional fractionation dose escalation for lung cancer.^10,11,32^ Isotoxic PBT dose escalation might play a role in re-irradiation in lung cancer (used in ≤30% of patients)^33^ and trials are on-going.

Prostate cancer was identified as potentially benefitting: while there is evidence of better tumour outcomes with higher doses,^34^ there is equipoise about the use of PBT and many authorities do not support PBT for prostate cancer.^11,12,35,36^ Breast cancer was also identified as potentially benefitting from dose escalation but this is not supported by the literature; reduced toxicity seems more likely to provide benefit.^11^ The prioritisation of anus/vulva in MoB 2 might be appropriate.^37^ However, clinical proof is awaited and skin toxicity, which has been highlighted recently,^38^ may reduce its advantage. These inconsistencies suggest a degree of optimism in the estimates which may not translate into reality. The relatively short clinical experience in the UK may also play a part. The subtleties of different indications within a single tumour site, such as pre-operative, post-operative or definitive radiotherapy, all of which may have different clinical benefits, are difficult to estimate without high quality clinical studies.

### Calculated MoBs 1 and 4

The percentages and absolute numbers from MoBs 1 and 4 are low (Table 3). For MoB 1, it is assumed that all patients under 16 having radical RT will benefit, except patients treated for leukaemia. Although only 0.5% of the total, this amounts to 97% of patients aged≤16 receiving radical RT.

The calculation for MoB 4 is the most uncertain because it requires a number of assumptions, especially on the latency of SMN and risk per unit dose of radiation. There was little effect from altering these values. Although MoB 4 is estimated to benefit only 0.1% of patients having radical RT, it will grow in importance as more patients are treated.

### Assumptions on the use of RT and of radical treatment

The most accurate predictions, of percentages and absolute patient numbers, are those derived from the NCRAS data. Interestingly, this produced slightly higher percentage estimates (13% vs  9%) but substantially lower absolute numbers than the generic values (Tables 2 and 3).

Compared to the 50%, 60% generic values, the NCRAS data showed considerably lower use of RT (31%, 70%). This has the effect of reducing the number of cases under consideration and raising the percentage benefit. Although the NCRAS data refer to treatments actually delivered, the numbers may not be ideal.

Additional ‘Mechanisms of Benefit’ , such as reduced immune-suppression, increased immune-activation, enhanced efficacy from combination with pharmaceutical agents, and potential with FLASH dose rates^2,39–41^ were not considered since there are as yet no clinical data to confirm benefit.

### Selecting patients for PBT

Selecting patients who will benefit most in an evidence-poor environment is a major challenge. The UK uses an indication list in adults covering the commonly accepted categories, with children and young adults under about 25 years of age eligible if they have curable tumours, reasonable expectation of 5-year survival and expected dosimetric advantage from PBT. Formal clinical studies will widen access for other patients. The UK is fortunate in having capacity to accommodate trials and flex the indications according to study results as they are published. Other countries, most notably the Netherlands, select some patients for PBT using NTCP modelling from comparative RT plans.^27,30,31,42^ Other solutions have also been proposed (*e.g.* ^43–45^) but use of dosimetry differences alone is not sufficient to predict clinical outcome.^11,13,46^

### Number needed to treat (NNTT)

It has been assumed that patients benefitting from PBT can be identified with 100% accuracy. Although comparative planning helps, there are several confounders including unusual anatomy, associated morbidity, and genetics.^47^ In effect, much larger numbers would have to be treated in order to maximise benefit, requiring greater capacity, while at the same time reducing the percentage of patients who actually express benefit.

### Patient numbers who might not benefit from PBT

There may be patients for whom there is neutral (no) benefit.^11,13^ This might include prostate cancer.^11,35^ Only a minority of patients who have dual planning and comparative NTCP calculations for HNC are selected for PBT, suggesting, at best, neutral benefit for some.^31^ There may also be patients who have better dosimetry with IMRT^48^ who might have either a neutral or disadvantageous outcome with PBT, particularly given the physical and biological uncertainties inherent in PBT.

### Additional considerations

This study highlights that there is a substantial lack of outcome data including comparison data between modern XRT and PBT. Systematic evaluation of PBT has been a key objective of the NHS from the outset.^1,49^ Formal clinical studies are already underway although they will need some years to mature. The first randomised trial opened in 2020,^29^ and others are following. The UK also has established other mechanisms for evaluation including evaluative commissioning, small registry studies and outcomes tracking for all patients treated in the UK.^4^ Pursuing level I randomised evidence in all groups may be unfeasible or unethical^14,15,50,51^ which supports this approach.^2,4,52–56^ Evolution of technology in both PBT and XRT makes evaluation even more challenging, especially for late effects. It is also important for funders to recognise that some endpoints, including but not only SMN, occur late after radiotherapy so that long-term follow up is essential.^11,53,57,58^

Technical, physical, computational and biological research and developments are needed to inform clinical studies and develop treatments, and will occupy the research communities for some time.^2,11,30,31,47,59–64^

## Conclusions

Less is known about the percentage of patients who may benefit from PBT than is generally acknowledged and expert opinion varies widely. Our consensus suggests that around 13% of patients treated with curative RT might benefit from PBT. This is likely to be an overestimate and a figure of 4.3% appears more secure. It remains unproven whether these are clinically realistic figures.

Considerable further work is needed to address this and the associated problem of the magnitude of benefit. Encouragingly, this is underway and includes trials, outcomes tracking of all patients receiving PBT, and international collaboration. The results suggest that the current NHS approach to commissioning, which is based on evidence and can expand when necessary, is a rational strategy.
